# The characteristics of cervical spinal cord trauma at a North Tanzanian Referral Hospital: a retrospective hospital based study

**DOI:** 10.11604/pamj.2019.33.82.18353

**Published:** 2019-06-04

**Authors:** Fanuel Damian Bellet, Sakina Mehboob Rashid, Mubashir Alavi Jusabani, Marieke Cornelia Johanna Dekker, Rogers Joackim Temu

**Affiliations:** 1 Department of Orthopedics and Traumatology, Kilimanjaro Christian Medical Centre, Moshi, Tanzania; 2 Department of General Surgery, Kilimanjaro Christian Medical Centre, Moshi, Tanzania; 3 Department of Internal Medicine, Kilimanjaro Christian Medical Centre, Moshi, Tanzania; 4 Department of Pediatrics and Child Health, Kilimanjaro Christian Medical Centre, Moshi, Tanzania; 5 Department of Neurology, Radboud University Medical Center, Nijmegen, Netherlands

**Keywords:** Cervical, traumatic, spinal cord injuries

## Abstract

**Introduction:**

Surviving a traumatic cervical Spinal Cord Injury (SCI) has an immense effect on an individual's physical function and independence. It also predisposes them to financial, social, psychological and several medical complications throughout their life. In high-income countries, improved multidisciplinary care has led to better long term outcomes, however in low-income countries, the burden of the condition and its associated mortality remain high. The aim of this study was to illustrate the sociodemographic and clinical characteristics of cervical level Traumatic Spinal Cord Injuries (TSCIs) at Kilimanjaro Christian Medical Centre (KCMC) in northern Tanzania.

**Methods:**

This was a retrospective hospital-based study of 105 cervical TSCI cases admitted to KCMC from January 2012 to December 2016.

**Results:**

We included 105 patients in the study cohort, with a male preponderance of 86.7%, giving a male-to-female ratio of 6.5:1. The mean age at injury was 44.1 years. Overall, 65.7% were farmers and 69 patients were from within the Kilimanjaro region. Road Traffic Crashes (RTCs) accounted for 47.6% of the injuries, 17.9% had associated injuries, 38.1% sustained complete TSCIs and 45.7% developed secondary complications during the ward stay. The mortality rate before discharge from hospital care was 35.2%.

**Conclusion:**

The majority of patients were males from a low socioeconomic background and the most common cause of injury was RTCs. The secondary complication rates and mortality rates before discharge from hospital care are high.

## Introduction

Distinguished by their etiology, Traumatic Spinal Cord Injuries (TSCIs) are a result of physical force directed towards the bony vertebral column. Trauma which results in a lesion of the cord is often blunt and accompanied by multiple injuries to the structures which surround it. Vertebral injuries, ligament tears and disk prolapses often complicate the clinical picture [1]. Trauma to the vertebrae and its supporting structures may be severe enough to disrupt its protective function to the spinal cord. The resulting crush injury, dislocation, unstable or stable fracture can result in a spinal cord lesion of varying form including; a contusion, crush injury, laceration, well defined transection or nerve root avulsion [1]. Spinal cord lesions in the C1-C3 region are the most taxing SCIs often leaving patients ventilator-dependent with limited communication and neck muscle control [2]. The description of functionality following high cervical cord lesions is in stark contrast to the near complete independence which may be recovered by individuals who sustain an injury in the L2-S5 regions [2]. Apart from the differing clinical experiences based on level of injury, subsequent to the analysis of costs incurred following admission, a drastic difference between tetraplegic patients and paraplegic patients is demonstrated [2]. A publication from the American National Spinal Cord Injury Statistical Center stated that for individuals injured at age 25, lifetime costs would amount to 4.6 million United States Dollars (USDs) for high tetraplegia while paraplegic patients would incur a much lower figure of USD 2.3 million [3]. Although the figures listed for the United States of America may not be comparable to expenditure in regions of the world with differing economic levels of development, they are a clear indication that tetraplegic patients face a tougher path to adapt and re-assimilate back into society. The cervical region of the vertebral column is the most vulnerable to trauma due to the relatively axial alignment of the facet joints between the bony vertebral bodies. Lesser force is required to dislocate cervical region facet joints compared to those in the thoracic and lumbar region. Additionally, the thoracic cage and abdomino-pelvic organs lend support to the thoracic and lumbar regions of the spine respectively [4].

The level of the lesion is a strong indicator of the mortality due to injury, risk of secondary complications, cost incurred during treatment and rehabilitation and the nature of rehabilitation which will be required [2]. Mortality following TSCIs has been analysed frequently by numerous studies and the impression of level of injury has been unmistakable; patients with tetraplegia are more likely to succumb to their injuries when matched with cohorts of patients with lesions below the cervical cord [2]. TSCIs at progressively higher cord levels are more likely to occur with concomitant head trauma resulting in a decreased level of consciousness during initial presentation to an acute care facility; thus likely contributing to a higher incidence of aspiration [5, 6]. Traumatic injury to the cervical region of the spinal cord has been associated with the development of dysfunctional swallowing - dysphagia - with an increased risk of aspiration which would contribute significantly to the incidence of pulmonary complications in the TSCI cohort with higher level injuries [7]. Rehabilitation and assimilation into society are challenges which require the input of multiple disciplines to ensure a satisfactory outcome for a patient; it goes without saying that the financial input required is an unwelcome addition to health care budgets of low- and middle-income countries. The physical disability associated with surviving a cervical TSCI greatly diminishes the quality of life of the individual. Article 10 of the United Nations Convention on the Rights of Persons with Disabilities states that 'state parties reaffirm that every human being has the inherent right to life and shall take all necessary measures to ensure its effective enjoyment by persons with disabilities on an equal basis with others' [8]. This paper aims to illustrate the characteristics of cervical TSCIs at a tertiary health facility in Tanzania. By contributing to existing literature which discusses SCIs in eastern Africa, we would like to build the ground work for the assessment of resources required in the primary prevention, acute management and satisfactory rehabilitation of cervical SCIs.

## Methods

**Setting and study population:** the study was conducted in the Department of Orthopedics and Trauma at KCMC. KCMC is one of Tanzania's four referral hospitals, located in the northern region of the country. The study period ranged from January 2012 to December 2016. The inclusion criteria for the study was all patients who were admitted to the KCMC Orthopedics and Trauma Ward from January 2012 to December 2016. Patients were excluded from the cohort if files could not be traced or if significant sections of the records were missing. The limited health care facilities at KCMC are a reflection of the country's strained health care budget; often only an x-ray of the spine can be afforded by the patient and computed tomography scans are subject to affordability on the patient's part and machine maintenance difficulties. The nearest magnetic resonance imaging scanners are available only in private clinics in Arusha city, over 80 kilometers away. Patients who are clinically stable for transport may still remain in the hospital due to scarcity of appropriate vehicles to transport patients, unavailability of trained personnel to handle TSCI patients and the danger of reckless driving on the highway connecting Moshi town and Arusha city. Patients who complete the acute management period in the Orthopedics and Trauma wards are transferred to the Orthopedic Rehabilitation Unit (ORU) for rehabilitation. KCMC's ORU is the only unit of its kind in the country which offers tailored in-patient rehabilitation services involving multidisciplinary care.

**Methodology and variables:** this was a retrospective hospital-based study. The admissions logs for the ward were utilized to compile a list of file numbers, followed by file retrieval from the Department of Medical Records. Patient admission and in-ward notes were methodically studied and required data were extracted detailing demographics, injury characteristics, ward progress and final outcome. Collected data were encoded, entered and analyzed by Statistical Package for the Social Sciences version 20 software.

## Results

**Study population:** a total number of 105 patients were included in the study cohort, with a male preponderance of 86.7% (n=91). The age range of the study cohort was 13 to 88 years with a mean age of 44.1 years. The most commonly affected age group was 16 - 30 years (n=29, 27.6%) (Table 1). An overwhelming 65.7% (n=69) of the cohort were farmers and most patients resided within the Kilimanjaro Region (n=59, 56.2%). Road Traffic Crashes (RTCs) accounted for most of the injuries (n=50, 47.6%) followed closely by fall injuries (n=43, 41.0%) (Table 2). A majority of the patients presented at KCMC within the first 24 hours of injury (n=61, 58.1%) and 38.1% (n=40) arrived more than 3 days later.

**Table 1:** Age distribution of the study cohort

Age (years)	n	%
0 - 15	2	1.9
16 - 30	29	27.6
31 - 45	27	25.7
46 - 60	28	26.7
61 - 75	15	14.3
>75	4	3.8

**Table 2:** Causes of cervical TSCIs in the study cohort

Cause of Injury	n	%
RTC	50	47.6
Car	28	25.9
Motorcycle	22	21.0
Falls	43	41.0
Above 1 meter	24	22.9
Below 1 meter	19	18.1
Other	12	11.4

**Injury characteristics:** radiographic examination demonstrated that lower cervical spine injuries were the most common (n=59, 56.2%) followed by upper cervical spine injuries (n=24, 22.9%). Eighteen (17.1%) x-rays demonstrated only features of degenerative joint disease or no abnormality and 4 (3.8%) demonstrated injuries at multiple skeletal levels.

***Associated injuries:*** although the sole injury in 82 (78.1%) patients was the cervical TSCI, associated head injuries were observed in 12 (11.4%), chest injuries in 3 (2.9%) and long bone fractures in 3 (2.9%).

***Severity of injury:*** complete spinal cord injuries were observed in 40 individuals (38.1%).

**Secondary complications:** following admission, the prevalence of secondary complications while in the ward was 45.7% (n=48) (Table 3). Pressure sores occurred most commonly (n=24, 22.9%); spasticity occurred in 22 individuals (21.0%) and respiratory complications in 17 (16.2%). Pressure sores frequently developed in the sacral region (n=43, 41.4%) and on the heels of patients (n=20.1, 19.7%).

**Table 3:** Incidence of secondary complications in the study cohort

Secondary Complications	n	%
Pressure sores	24	22.9
Spasticity	22	21.0
Respiratory complications	17	16.2
UTI	15	14.3
GI complications	12	11.4
Neuropathic pain	9	8.6
Contractures	4	3.8
Neurogenic bladder	3	2.9
Autonomic dysreflexia	3	2.9
Other	2	1.9

**Mortality:** the mortality rate before discharge from hospital care of the study population was 35.2% (n=37). A higher rate of mortality was associated with complete TSCIs (70%) and lower cervical TSCIs (42.4%); most deaths occurred within a week of admission (n=67, 63.9%).

## Discussion

The recurrence of a preponderance of male patients in TSCI populations across several studies cannot go unnoticed [9]. As the pattern frequently appears after the pediatric age group, it is a reflection of the manner in which gender roles affect the risk exposure of the general population. Literature from Africa has reported male to female ratios of up to 12:0 [10]. Being a farmer by profession in Tanzania denotes a seasonal income dependent on rainfall. Additionally, in accordance with the culture in rural Tanzania, individuals with no formal employment are called farmers and it is largely small scale subsistence farming which is practiced by this group. Hospital records demonstrated that a large proportion of the study population (65.7%) were farmers. When viewed in light of the most affected age-group, 16-30 years (n=29, 27.6%), the loss of productivity is overwhelming. Patient transfer to hospital from the site of injury or health care center of primary contact to KCMC is repeatedly delayed and often in the back seat of a passenger car by untrained individuals; frequently on unpaved roads or tarmac roads which are poorly maintained. The interplay of the effects of nonexistent emergency medical care at the site of injury, poor transport practices and prolonged duration from injury to admission worsen the primary injury with negative implications on the prospect of recovery and rehabilitation. Patients are recurrently transferred in unsteady positions by individuals who aren't trained to nurse patients with cervical TSCIs.

Often, epidemiological data detailing the mechanism of injury reflects cultural norms and the social environment allowing identification of high risk situations for injury. A Turkish study identifying falls as a major cause of TSCIs linked the statistics to seasonal incidences of injury and concurred that individuals who sleep on rooftops during the summer months are at an increased risk of sustaining a TSCI [11]. Researches conducted in Afghanistan - a conflict ravaged country - identified war-wounds as the primary cause of TSCIs [11]. Brazil (42%), Turkey (25%) and South Africa (21%) report amongst the highest proportions of TSCIs attributable to violence related incidents [11]. From KCMC, a publication dating back to 1985 detailed the clinical course of 47 patients with paraplegia admitted following traumatic incidents; 19 (40.4%) had been injured due to a fall from a tree and 11 (23.4%) had been involved in RTCs [12]. The higher incidence of injuries resulting from RTCs in the current study group is likely due to increased motor traffic levels since the 1980s. Although the outcome of TSCIs depends largely on the level and completeness of the lesion, awareness of the circumstances and contributing factors associated with TSCIs allows assertion of the most effective targets for primary prevention. The authors would have preferred to retrospectively collect data detailing the neurological level of injury however, this record was often missing from the admission and ward progress notes. Only a minority of patients had a recorded neurological level often with no record of progression of the lesion over subsequent days in the ward. Admission and daily ward round notes are often recorded by intern doctors who may not be fully conversant with a detailed neurological examination. Additionally, the Department of Orthopedics is often understaffed and patients who are deemed as 'stagnant' cases may not receive as much attention as individuals with open fractures, septic wounds requiring debridement in an operating theatre, fractures of the long bones or those requiring high care following surgery [13].

Often muscle strength would be recorded as a single value for each limb and progressive ward round notes would only document progress over time with vague references to improving or plateaued muscle power. References to specific myotomes was scarce upon admission. In an effort to ensure that this vulnerable TSCI population receive the highest standard of care possible, since the initiation of the multidisciplinary ward rounds at KCMC, every patient's neurological examination is conducted in accordance to the International Standards for Neurological Classification of Spinal Cord Injury and duplicate records are maintained; in the patient's file as well as a secure hospital registry of TSCI patients [14]. This has allowed a better assessment of the patient's progress and the provision of more personalized rehabilitation services. It was possible to review imaging for every patient included in the study to allow assessment of the skeletal level of injury. For the purposes of this study, the C1-C4 vertebrae were classified as the upper cervical spine and the C5-C8 vertebrae as the lower cervical spine. The brainstem structures which control the central cardiorespiratory drive are in close proximity to the levels of the cervical cord which innervate the diaphragm, C3-C5. The high risk of mortality at the site of trauma following injury to these structures may explain why only 22.9% of the patients in the study cohort were C1-C4 level injuries. Patients with high quadriplegia will remain dependent for a majority of activities of daily living while individuals with injuries in the C5-T1 region will be able to accomplish a variable number of these activities with complete independence or assistive technology depending on the exact level of the lesion [2]. Patients with associated injuries numbered 23 (21.9%), head injuries being the most common (n=12, 11.4%). Individuals who sustain both head injuries and cervical spinal cord injuries are at increased risk of cardiorespiratory arrest; central cardiorespiratory drive from the brain steam and innervation of the diaphragm can both be disrupted during the trauma.

KCMC management protocols require initial admission to General Surgery Wards if the patient has sustained both a severe head injury and TSCI. This translates into a possibility that the study population may be an under representation of the cervical TSCIs in the hospital. Additionally, the challenge of a complete nervous and musculoskeletal system assessment in a semiconscious or unconscious head injury patient denotes the potential of missing a cervical TSCI. Secondary complications occurred in almost half the patients who were admitted following injuries. It is interesting to note that certain complications do not arise at the site of injury but rather they are due to dysfunctions of distant physiological systems with disrupted nervous system control. Vigilance on the part of the management team cannot be emphasized enough since evidence indicates that with appropriate care and precautions, a number of these secondary conditions are preventable [15]. An analysis of the data demonstrated that pressure sores were the most commonly encountered secondary complication (n=24, 22.9%). Systemic reviews of publications from developing countries analyzed pooled data and determined a pressure ulcers prevalence rate of 26.7 - 46.2% in TSCI cohorts [16]. Of note is that the cited manuscript pointed out the likelihood that the figures are probably an underrepresentation of reality as most studies were conducted in low-resource settings with a limited capacity of reaching all individuals with TSCIs and inclusion criteria varied across the publications [16]. Reported to occur in an average 30 - 40% of patients following TSCIs, lesions result when skin or underlying tissue is injured following continuous pressure application or pressure combined with a shearing force [17]. Pressure ulcers can rapidly evolve from minor skin infections to potentially life threatening full-thickness wounds with tissue sloughing resulting in bone exposure. TSCI patients are uniquely susceptible to the development of pressure sores as they are often initially bed ridden, with diminished or absent sensitivity, fecal and urinary incontinence contributing to incessant moisture exposure, atrophying muscles and usually poor nutrition - all risk factors for the development of lesions [17].

The mortality rate before discharge from hospital care was 35.2% in the cohort; a majority of the deaths (70%) occurred in patients with complete TSCIs. A complete TSCI entirely disrupts the physiological continuity of the spinal cord and leaves the region distal to the point of the lesion devoid of higher center control. Considerable differences in the counts of mortality have been noted when patients with complete TSCIs are compared to their counterparts with incomplete lesions [15]. Following the comparison of mortality rate data from developing and developed countries, the differences are starkly obvious and are likely due to the interplay of several factors which come into play from transport of the injured to the hospital, acute care and the occurrence and management of secondary complications [18, 19]. Although KCMC's multidisciplinary spine team attends to all patients in the hospital with a SCI, acute management of SCI patients is often challenging due to delayed presentation to the hospital and the nonexistence of neurosurgical services at our center. Limited expertise and lack of required equipment has translated into limited experience with spine surgery at KCMC. Cervical TSCIs are a significant threat to public health, even more so in an era with increasing road traffic levels [20]. They also represent a challenge to every aspect of health care due to the multi-disciplinary and labor-intensive approach required to manage and rehabilitate patients. Living with a disability in Africa, where even access to basic health care is a challenge for the majority, translates into a poor quality of life and isolation from society. Apart from investing in primary prevention of these injuries, there is a need for every individual involved in TSCI care in Africa to look beyond the 'confines' of low resources and to focus on maximizing efficiency with available assets.

## Conclusion

The average cervical TSCI in-patient profile at KCMC is a young male from an economically fragile background. A strained health care budget in a low-income country also manifests in the high secondary complication rate. Cervical TSCI patients in a low resource setting have a grim prognosis, further compounded by the social and financial implications of the acquired disability.

### What is known about this topic

- Cervical TSCI's represent a burden to healthcare systems globally and especially so in low-income countries with limited medical resources;
- Cervical TSCI's require a multidisciplinary approach to aid recovery and guide rehabilitation;
- KCMC is home to the only spinal cord injury rehabilitation unit in the country.

### What this study adds

- The average patient with a cervical TSCI in northern Tanzania is a middle aged male, from a low socioeconomic background;
- A strained healthcare budget in a low-income country also manifests in the high secondary complication and mortality rates in individuals who have sustained a TSCI.

## Competing interests

The authors declare no competing interests.
